# Associations between regular physical exercise and physical, emotional, and cognitive health of older adults in China: an 8-year longitudinal study with propensity score matching

**DOI:** 10.3389/fpubh.2024.1301067

**Published:** 2024-04-09

**Authors:** Xiaoyan Xu, Yawen Zheng, Juan Fang, Jiahui Huang, Xudong Yang, Xianghe Zhu, Yanlong Liu, Li Chen, Shaochang Wu

**Affiliations:** ^1^Department of Psychiatry, The Third People’s Hospital of Huzhou Municipal, The Affiliated Hospital of Huzhou University, Zhejiang, China; ^2^School of Mental Health, Wenzhou Medical University, Wenzhou, China; ^3^Lishui Second People's Hospital Affiliated to Wenzhou Medical University, Lishui, China; ^4^Department of Psychology, School of Mental Health, Key Laboratory of Alzheimer’s Disease of Zhejiang Province, Institute of Aging, and Zhejiang Provincial Clinical Research Center for Mental Disorders, The Affiliated Kangning Hospital, Wenzhou Medical University, Wenzhou, China; ^5^Oujiang Laboratory (Zhejiang Lab for Regenerative Medicine, Vision and Brain Health), Wenzhou, China

**Keywords:** daily living activity, depressive symptoms, cognitive decline, physical exercise, propensity score, older adults

## Abstract

**Background:**

The importance of healthy aging is growing in China as it has the largest number of older adults in the world and is one of the fastest-aging countries. This study aimed to examine the predictive value of regular physical exercise in relation to the physical, emotional, and cognitive health among samples of adults aged ≥60 years in China during an 8-year period.

**Methods:**

A total of 10,691 older adults were extracted from two waves of national data from the China Family Panel Studies in 2010 and 2018. To minimize the impact of selection bias on the findings, a longitudinal propensity score matching (LPSM) method was used to examine the relationships between regular physical exercise and emotional health (depression), between regular physical exercise and physical health (instrumental activities of daily living), and between regular physical exercise and cognitive health (cognitive ability) of older adults. After LPSM, 856 older adults were included in the study. In the regular physical exercise group, the average age of participants at baseline year was 65.67 years, with an average age of 65.90 years for 238 men and 65.45 years for 190 women, and in the non-physical exercise group, their average age at baseline year was 65.70 years, with an average age of 65.45 years for 253 men and 65.98 years for 175 women.

**Results:**

LPSM indicated that regular physical exercise has been found to be effective in improving physical function and reducing depressive symptoms in old adults, even after controlling for background differences. However, the sensitivity analysis suggests that the positive association between regular physical exercise and cognitive function may not be sufficiently valid.

**Conclusion:**

The findings of this study indicate that engaging in long-term structured and repetitive physical exercise can have a significant positive effect on reducing depressive symptoms and improving the physical function of older adults. As a result, incorporating regular physical exercise into the lifestyle of older adults is recognized as an effective strategy for promoting healthy aging and reducing the strain on public health resources.

## 1 Introduction

Population aging is considered a significant achievement for humanity, but it also presents significant challenges. China, with the largest number of older adults in the world, is one of the fastest-aging countries (1). By 2021, China had approximately 267 million people aged 60 years and above. However, like many other nations facing the fast-growing aging population for the first time in history, the gap in the literature regarding the promotion of sustainable good health-related quality of life in aging populations is hindering the advancement of China toward healthy aging, an important global issue that warrants immediate investigation (2).

Aging is a progressive process that leads to the deterioration of various physiological functions in organisms (3). One of the significant declines observed from midlife to old age is the gradual decrease in physical health (4–6). This decline can result in the loss of physical independence, an increased risk of falls, and a diminished quality of life (7–9). Mental health problems are also common among older adults (10, 11). It is estimated that at least one in four older adults experience mental disorders such as depression, anxiety, or dementia, and this number is expected to double by 2030 (12–14). In addition, cognitive decline is a common health issue among older adults. According to a CDC report in the United States, adults aged over 64 years have a higher prevalence of subjective cognitive decline (11.7%) than adults aged 45–64 years (10.8%) (15). A systematic review published in 2021 found that the prevalence of mild cognitive impairment (MCI) among Chinese community-dwelling populations over 55 years old was 12.2%, and it increased with age (16). In conclusion, there is a pressing need to identify effective strategies that can support healthy aging and maintain or enhance physical, emotional, and cognitive health in older adults.

Regular physical exercise is a major contributor to healthy aging, offering a low-cost, low-risk, and effective strategy for addressing deteriorating health conditions in older adults (17–19). Numerous epidemiological studies have consistently demonstrated the positive effects of regular physical exercise on the human body. These effects include reducing frailty, improving muscle strength and endurance, lowering body mass index (BMI), and decreasing the risk of major mortality factors. Regular physical exercise has also been found to extend life expectancy in patients with arterial hypertension (20), diabetes mellitus type 2 (21), dyslipidemia (22), coronary heart disease (23), stroke (24), or cancer (25). Additionally, research consistently indicates that regular physical exercise has positive effects on emotional health (26, 27), with a particular focus on reducing depressive symptoms (28, 29) and preventing the occurrence of depression (30, 31). Exercise plays a role in regulating neurotransmitter levels, such as serotonin and dopamine, which are essential for mood regulation (32). It also promotes neurogenesis and neuroplasticity, enhancing mood stability (33). In addition, exercise has anti-inflammatory effects and reduces stress hormones, which contribute to alleviating depression (34). The release of endorphins during exercise acts as a natural mood elevator. Furthermore, cognitive distraction and social interaction during physical activity also play roles in improving mental wellbeing (35). Overall, scientific evidence highlights that exercise provides a holistic approach to mitigating depressive symptoms and enhancing mental health. Inconsistent findings also have been reported regarding the associations between regular physical exercise and cognitive functions (36). However, some studies have shown a positive correlation between regular physical exercise and the maintenance or enhancement of cognitive function (37, 38). Other studies have reported a dose–response relationship (39–41) or found no significant correlation (42).

While there are numerous benefits of regular physical exercise on the emotional, physical, and cognitive health of older adults, several important challenges still persist.

The first significant challenge is that many studies examining the impact of regular physical exercise on emotional, physical, and cognitive health in older adults have small sample sizes, which limits their ability to demonstrate strong effects. For instance, a systematic review of 14 randomized controlled trials focused on the impact of exercise on cognitive outcomes in adults with mild cognitive impairment. The review revealed that most of the trials had small sample sizes (*n* < 200), which restricted their ability to detect small effects. Only 8% of the cognitive outcomes were statistically significant. These findings suggest a lack of strong and consistent evidence supporting significant and robust cognitive improvement through exercise in individuals at risk of dementia (43). Second, regular physical exercise recommendations for health are generally based on studies with a cross-sectional design or a short-term longitudinal design using young or middle-aged adults or clinical populations. Finally, selection bias is a significant concern in studies on regular physical exercise. It is widely recognized as one of the most substantial biases that can impact the findings of empirical studies (44). The existing literature suggests that various demographic and socioeconomic variables are associated with the health of older adults (45, 46). These variables include gender, age, education level, marital status, household registration, income, interpersonal relationships, chronicity, and the number of children. For instance, men and women exhibit physiological and cultural differences that contribute to disparities in health outcomes (46, 47). Age also plays a significant role, as the immune system weakens with age, resulting in a decline in both physical and mental health (45, 46). Higher education levels and being married are protective factors for health. Moreover, urban older adults in China generally experience better health than their rural counterparts (48–51). Having a higher income or good interpersonal relationships also promotes better health (52, 53). Chronic illnesses can directly harm an individual’s physical health and lead to negative emotions (54). Finally, the number of children can influence the economic and social behavior of parents, which subsequently affects the health of older adults (55). Therefore, these natural differences between older adults who do regular physical exercise and those who do not will affect the external validity of the analysis and lead to unreliable research outcomes.

To address these challenges, the present study aimed to further examine the predictive value of regular physical exercise in relation to physical, emotional, and cognitive health. To address confounding factors and selection bias, we conducted a longitudinal propensity score matching analysis (LPSM). The selection of suitable confounding variables is the basis for applying LPSM. Based on the literature review, we identified more than 10 confounding variables between regular physical exercise and physical, emotional, and cognitive health in older adults. To generate national results, we focused on older adults aged ≥60 years using data from the China Family Panel Studies (CFPS), which is a comprehensive, longitudinal social survey covering a wide range of social phenomena in contemporary China (56). Finally, we analyzed the long-term longitudinal effect of regular physical exercise using matched data from the 2010–2018 waves of CFPS.

This study utilized CFPS data to assess the impact of regular physical exercise on the physical, emotional, and cognitive health of older adults aged ≥60 years in China over an 8-year period. The longitudinal propensity score matching analysis (LPSM) method was employed to minimize the influence of potential confounding factors.

## 2 Method

### 2.1 Participants

The data used for this study was gathered from the CFPS of the years 2010 and 2018. The CFPS is a longitudinal research project administered by the Institute of Social Science Survey (ISSS) at Peking University, China. A multi-stage probability-proportional-to-size (PPS) strategy with implicit stratification was performed in the sampling process that comprises three stages: the county level as the primary sampling unit, a community or village for the second-stage sampling unit, and household as the final sampling unit. CFPS surveyed respondents in sampling units in 25 provinces and consisted of a rich set of socioeconomic questions and information on the levels of individual, family, and community. Details of the original study have been reported elsewhere (56, 57). The Peking University Biomedical Ethics Review Committee provided ethical approval of the survey (approval number: IRB00001052-14,010). Respondents were given a statement explaining the purpose of the study, and all study participants signed a written informed consent prior to being investigated. All methods will be carried out in accordance with relevant guidelines and regulations of the Declaration of Helsinki.

**Figure 1:**
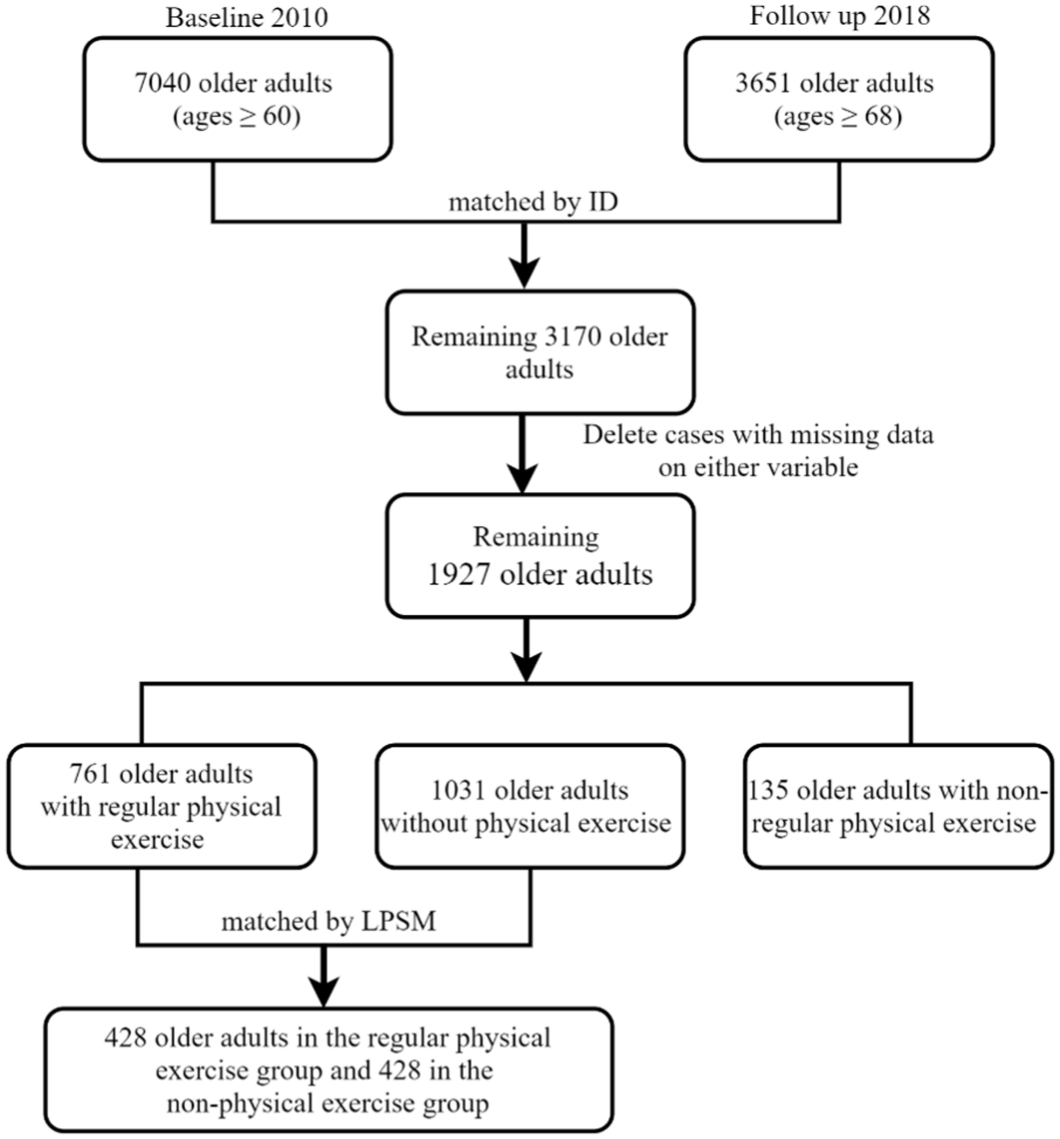
Construction of panel data. Source: CFPS (65). China Family Panel Studies (CFPS) is a nationally representative, biannual longitudinal survey of Chinese communities, families, and individuals launched in 2010 by the Institute of Social Science Survey (ISSS) of Peking University, China. CFPS (65) means 2 waves of the China Family Panel Studies data in 2010 and 2018.

Following an initial baseline survey wave in 2010, ISSS conducted four follow-up survey waves in 2012, 2014, 2016, and 2018. For the purposes of the survey, first, we selected 7,040 older adults who were at or over 60 years of age from the 2010 data of CFPS and 3,651 older adults who were 68 years old or older from the 2018 data of CFPS. In total, 3,170 subjects remained by matching ID in 2010 and 2018, and subjects with missing data on relevant variables in this study were deleted. As a result, 1,927 subjects were left. Second, among them, 761 older adults were with regular physical exercise, 1,030 older adults were without physical exercise, and 135 older adults with non-regular physical exercise were not included in this study. Third, by the method of LPSM, 761 older adults in the regular physical exercise group and 1,031 older adults in the non-physical exercise group were matched on 12 covariates, and 428 subjects remained in each group finally (see Figure 1 for the sample selection).

### 2.2 Eligibility criteria

Participants meeting the following inclusion criteria were included in this study: (a) individuals aged 60 years or above in 2010; (b) individuals who participated in both the 2010 and 2018 waves; and (c) individuals with no missing data on relevant variables. In the 2010 CFPS data, there were 7,040 older adults aged 60 years and above, who would be 68 years and above in 2018. In the 2018 CFPS data, there were 3,650 older adults aged 68 years and above, of which 3,170 had also participated in the 2010 CFPS. After excluding cases with missing data in any relevant variable, a total of 1,927 subjects remained. Among them, 761 older adults were in the regular physical exercise group, 1,031 older adults were in the non-physical exercise group, and 135 older adults with non-regular physical exercise were not included in this study. Using the LPSM method, 761 older adults in the regular physical exercise group and 1,031 older adults in the non-physical activity group were matched on 12 covariates, resulting in 428 subjects remaining in each group (see Figure 1 for the sample selection).

### 2.3 Procedures and measures

#### 2.3.1 Physical health

The physical health of older adults was evaluated by the instrumental activities of daily living (IADLs), which were confirmed to be a core set of essential activities for individuals to live independently and the key measures of physical health for older adults in the existing studies (58–60) IADLs in CFPS were measured by a modified version of the Lawton IADLs Scale (61). Respondents were asked whether they could perform these seven tasks without assistance: (a) outdoor activities (e.g., walking 300 m); (b) kitchen chores (e.g., preparing meals and washing dishes); (c) shopping for groceries; (d) having meals; (e) managing transportation; (f) housekeeping (cleaning); and (g) doing laundry. A binary variable was constructed for IADLs, with 0 representing difficulty with any of the seven IADLs and 1 representing “no” difficulty with any of the seven IADLs. Previous research has demonstrated that the Chinese version of the IADLs scale exhibits strong internal consistency (coefficient alpha = 0.86) and good test–retest reliability (*r* = 0.90) (62). The IADLs Scale in CFPS has proven to be a valid measure of the daily performance of older adults (63, 64). Measurement of IADLs functions is essential as it serves as an early sign of functional decline in old age. These measurements are also predictors of the need for alternative living arrangements, the utilization of paid home care, and admission to nursing homes (64). In the current sample, the IADLs Scale shows good internal consistency (coefficient alpha = 0.83).

#### 2.3.2 Cognitive health

The cognitive health of older adults was evaluated by cognitive ability tests in CFPS (56). The theoretical basis of the CFPS cognitive ability tests was the design of the Guttman Scale in psychometry, which showed good reliability and validity (66). Cognitive ability tests in CFPS applied both vocabulary and mathematics tests as measurement tools to measure and represent “crystallized intelligence,” which is referred to as acquired knowledge through learning, experience, and education (56, 67). Previous research has shown that the vocabulary and mathematics tests in CFPS have strong internal consistency (coefficient alpha = 0.85–0.96) and criterion validity (r = 0.80–0.86) (68). These tests have been widely used and validated for measuring cognitive ability in Chinese adults and adolescents in previous studies (66, 69–72).

The vocabulary test consists of 34 word-recognition questions, which measure one’s vocabulary by how difficult of a character he or she can recognize and are sorted in ascending order of difficulty. The respondents were asked to recognize the increasingly difficult characters one by one until they failed to recognize three consecutive characters. The final test score was based on the rank order of the last character recognized by each respondent, ranging from 0 (lowest) to 34 (highest).

The mathematics test consists of 24 mathematical questions. The procedures for the mathematics test were similar to those mentioned above. The test score was assigned using the same rank-order rule as that in the vocabulary test and recorded on a scale from 0 (lowest) to 24 (highest).

#### 2.3.3 Emotional health

Depression, one of the most prevalent emotional problems among older adults, serves as an indicator of emotional health. In the 2010 and 2018 CFPS, depression was evaluated by the simplified version of the Centre for Epidemiologic Studies Depression Scale (CESD) (73): Six questions measure depressive mood: (a) I felt depressed; (b) I felt it was very hard to do anything; (c) My sleep was restless; (d) I felt lonely; (e) I felt sad; and (f) I felt that life could not continue; and two questions measure positive mood: (g) I was happy and (h) I enjoyed life. These items have four answer options: almost none (less than 1 day), sometimes (1–2 days), often (3–4 days), and most of the time (5–7 days). The corresponding score of each option is 1, 2, 3, and 4. The higher the total score is, the more severely depressed the subjects feel. CESD-8 is a widely used measure for screening depression in large-scale surveys among older people (74). It offers several advantages, including brevity, accessibility, and ease of use by non-mental health professionals in non-clinical settings. Additionally, it has demonstrated reliability and validity compared to longer scales (74). In the Chinese context, the CESD-8 has been extensively utilized and validated as a valuable and dependable tool for identifying the risk of depression among the older population, aiding in further diagnosis (75–77). Cronbach’s alpha of the study for the CESD-8 was found to be 0.85 and 0.81 for the 2010 and 2018 data, respectively.

#### 2.3.4 Regular physical exercise

According to the previous study (78–80), regular physical exercise was defined as planned, structured, and repetitive physical activity with the ultimate or intermediate goal of improving or maintaining physical fitness, and the frequency and duration of exercise were usually chosen to reflect the physical activity status of the population.

In the CFPS, physical exercise includes walking, long-distance running, jogging, and hiking; martial arts such as taijiquan and qigong exercises; and indoor and outdoor activities such as dancing, aerobics, gymnastics, and yoga, as well as ball sports with small and large balls, water sports such as swimming, diving, boating, and sailing, winter ice and snow sports, and contact sports such as wrestling, judo, and boxing. The following two questions were used in the CFPS to determine whether older adults do regular physical exercise (56): (a) Regarding the frequency of physical exercise, respondents were asked, “How often do you participate in physical exercise?” Responses were categorized into the following seven levels: never; less than 1 time per month on average; more than 1 time per month on average, but less than 1 time per week; 1–2 times per week on average; 3–4 times per week on average; 5 or more times per week on average; and 1 time a day; and (b) regarding the duration of physical exercise, respondents were asked, “In the past 12 months, how many minutes did you exercise at a time?” As such, this variable is also a continuous variable. With reference to previous studies on the division of regular physical exercise and the answers to the above two questions, we divided the participants into two groups: (a) regular physical exercise group (participants engaged in physical exercise “at least three times a week” and at least 30 min per time in two waves of CFPS surveys in 2010 and 2018) and (b) non-physical exercise group (participants who indicated that they “never” do any physical exercise in two waves of CFPS surveys in 2010 and 2018).

#### 2.3.5 Covariates

Based on the previous studies (46, 47, 51, 81, 82) and information in the 2010 and 2018 CFPS, this study initially identified 12 potential confounding covariates: gender, age, number of children, educational status, household registration, marital status, income level, chronic diseases, interpersonal relationships, physical (2010), depression (2010), and cognitive (2010) health. Educational status included the following: 1—illiteracy, 2—elementary school, 3—junior high school, 4—high school/vocational high school, 5—junior college, 6—university undergraduate, 7—master, and 8—doctorate; household registration included the following: 0—rural and 1—urban; marital status included the following: 0—having no spouse and 1—having a spouse; income level was measured by one question: How do you perceive your income level in local locations? (1—very low, 5—very high); chronic disease included the following: 0—having no chronic disease and 1—having at least one chronic disease; and interpersonal relationships were measured by one question: How do you perceive your interpersonal relationships? (0—worst; 10—best).

### 2.4 Statistical analysis

In this study, LPSM is the key statistical technique used to effectively eliminate sample-selection bias and mixed bias by matching the individuals in the treatment group with the comparable objects in the control group (83, 84). LPSM analyses are generally divided into two steps (83): The first analysis is used to calculate the propensity score and match subjects according to the propensity score. The propensity score estimated by the logit regression model was employed to match each subject between the regular physical exercise and non-physical exercise groups in similar conditions, and it is estimated as follows:

$$P=P\left(D=1|x\right)=\frac{1}{1+e^{-\beta_{0}-\sum \beta_{i}x_{i}}}$$

Where P represents the probability of subjects participating in regular physical exercise, D = 1 represents subjects in the regular physical exercise group, and x refers to 12 covariates.

The second analysis is used to estimate the average treatment effect on the treated (ATT) by the following model, which reflects the effect of regular physical exercise on the emotional, physical, and cognitive health of older adults. The significance of ATT is tested by a paired *t*-test (85).

$$ATT=E\left(Y_{1}|\;D=1\right)-E\left(Y_{0}\;|\;D=1\right)=E\left(Y_{1}|\;D=1,\;P\right)-E\left(Y_{0}\;|\;D=0,\;P\right)$$

Where Y_1_ and Y_0_ are the dependent variables of the matched samples in the regular physical exercise and non-physical exercise groups, respectively, P is the propensity value, D = 0 indicates the subjects without PE, and D = 1 indicates subjects with the regular physical exercise. As Y_0_|D = 1 cannot be directly observed, the establishment of the above model must satisfy the “unconfoundedness assumption,” which requires control factors associated with the participation of older adults in regular physical exercise. Finally, a boundary sensitivity analysis was conducted to explore to what extent the hidden selection bias can change the results of the treatment effectiveness obtained (84).

Covariate screening was conducted using the correlation analysis. To compare differences between the regular physical exercise group and the non-physical exercise group, the independent-sample *t*-test and the chi-squared test were employed. The significance level for all tests was set at 0.05. PSMATCH2 and NNMATCH in Stata 14.0 were performed to conduct the statistical analysis.

## 3 Results

### 3.1 Covariate screening

Covariates in LPSM should include factors that are related to both independent and dependent variables in order to obtain a net estimate of the effect of independent variables on dependent variables (83, 86). To screen out the covariates that are related to regular physical exercise, physical health, emotional health, and cognitive health (2018), the correlation coefficients were calculated, respectively. The results are presented in Supplementary Table S1. The level of regular physical exercise was found to be significantly related to 10 covariates, except for marital status (*p* = 0.16) and chronic disease (*p* = 0.07). However, these two covariates are significantly related to physical (*p* < 0.01), emotional (*p* < 0.01), and cognitive health (*p* < 0.01). Therefore, to investigate the association between regular physical exercise of older adults and the three health factors using the LPSM method, all of the aforementioned covariates should be included.

### 3.2 Descriptive statistics

Descriptive analyses were conducted in this study to examine two different types of samples and compare differences between two groups of samples. The study included 761 older adults who engaged in regular physical exercise and 1,031 older adults who did not engage in physical exercise. Supplementary Table S2 shows that there were significant differences in 12 covariates, except for marital status, between the two groups. Compared to the non-physical exercise group, the regular physical exercise group had a higher education status, a higher income level, more number of children, better interpersonal relationships, and other factors. These significant differences should be taken into account when evaluating the relationships between regular physical exercise and the emotional, physical, and cognitive health of older adults.

### 3.3 Propensity score matching analysis

A logistic regression model was conducted to analyze the relationship between regular physical exercise and 12 covariates. The results of the regression showed that the model had a strong overall explanatory power. Among the covariates, age (*p* = 0.02), household registration (*p* < 0.01), income level (*p* < 0.01), interpersonal relationships (*p* < 0.01), IADLs in the 2010 year (*p* = 0.03), depression in the 2010 year (*p* < 0.01), and cognitive ability in the 2010 year (*p* < 0.01) were found to be significant predictors of regular physical exercise among older adults (refer to Table 1). The regression results would be used to build a prediction model to calculate the propensity of an older adult participating in the regular physical exercise. The higher the propensity, the more likely the older adults do the regular physical exercise.

**Table 1:** Logistic regression estimates of regular physical exercise among older adults (*N* = 1792).

Variables	OR	Std. error	*Z*	*P*	[95% Confidence Interval]
Constant	0.005	0.006	−4.490	0.000	[0.000,0.049]
Gender	0.896	0.118	−0.830	0.405	[0.693,1.160]
Age	1.032	0.014	2.350	0.019	[1.005,1.059]
Educational status	1.017	0.048	0.360	0.715	[0.928,1.115]
Household registration	5.510	0.722	13.030	0.000	[4.263,7.122]
Marital status	0.752	0.115	−1.860	0.063	[0.557,1.015]
Number of children	1.019	0.046	0.420	0.677	[0.933,1.113]
Income level	1.136	0.056	2.600	0.009	[1.032,1.251]
Chronic disease	1.024	0.132	0.180	0.856	[0.796,1.317]
Interpersonal relationships	1.104	0.032	3.470	0.001	[1.044,1.167]
Physical health ^a^	1.165	0.084	2.120	0.034	[1.012,1.343]
Emotional health ^a^	0.615	0.063	−4.720	0.000	[0.503,0.753]
Cognitive health ^a^	1.043	0.006	6.780	0.000	[1.030,1.055]
Pseudo *R*^2^	0.307				
ROC	0.833				

“a” represents the data of the variable from 2010 and the others from the data from 2018.

### 3.4 Matching and balanced tests

The calculated propensity was used to match 761 older adults with regular physical exercise and 1,031 older adults without PE. The nearest-neighbor matching method (1:1) was used, and finally, a total of 428 pairs of samples were successfully matched. As shown in Supplementary Table S3, the independent-sample *t*-test of each covariate between the control group and the treatment group was no longer significant (*p* > 0.05) after matching, which met the relevant recommended indicators.

**Figure 2:**
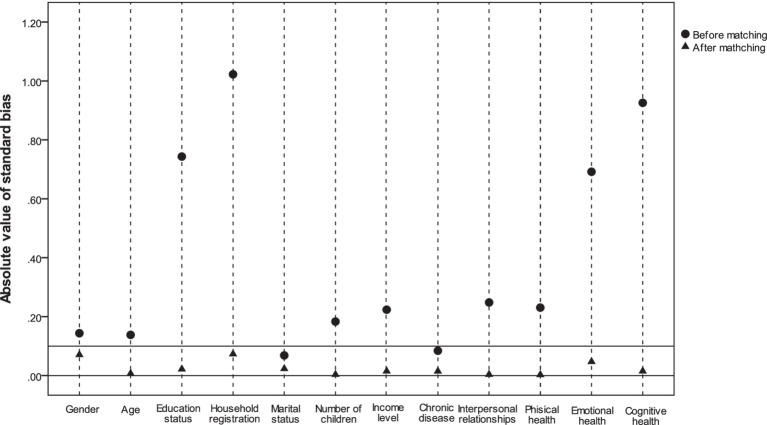
Changes in the absolute value of the standard deviation of the covariates before and after matching 12 covariates.

In addition, to specifically demonstrate how the covariate balance between the two groups was improved by propensity score matching, the absolute values of the standardized differences before matching and after matching are displayed in Figure 2. The standard deviations (Figure 2, triangles) of all covariates after matching were improved substantially, and the absolute values of the standard deviation of all covariates were below 0.1, indicating that the assumption of the covariate balance is satisfied.

### 3.5 Average treatment effect on the treated

In this study, the bootstrap method was used to estimate the average treatment effect on the treated (ATT) and empirical standard error after propensity score matching. The estimated results show that older adults with regular physical exercise (treatment group) showed significantly lower depression levels (*p* < 0.01), higher physical health (*p* < 0.01), and higher cognitive health (*p* < 0.01) than older adults without PE (control group) after the bias of confounding factors was eliminated by LPSM. The depression level was significantly reduced by 0.187, and physical health and cognitive health were significantly improved by 0.542 and 1.032, respectively (see Table 2). In general, compared with the non-PE group, the physical health and cognitive health of older adults in the regular physical exercise group can be improved by 8.963 and 5.029%, respectively, and the depression level was reduced by 10.47%.

**Table 2:** ATT of regular physical exercise on health of older adults.

Dependent variable	Regular physical exercise group	Non-physical exercise group	ATT	Bootstrap SE	*Z*	*P*
Physical health	6.589	6.047	0.542	0.078	6.949^**^	0.000
Emotional health	1.599	1.786	−0.187	0.028	−6.679^**^	0.000
Cognitive health	21.846	20.799	1.032	0.401	2.573^*^	0.01

(1) SE—standard error; ATT—average treatment effect on the treated; (2) ^*^p < 0.05, ^**^p < 0.01, ^***^p < 0.001.

### 3.6 Sensitivity analysis

To further explore to what extent the hidden selection bias can change the results of the treatment effectiveness obtained, a sensitivity analysis with Rosenbaum bounds was used to assess the robustness of the estimates. Rosenbaum’s method of sensitivity analysis relies on the sensitivity parameter gamma, which was defined as log odds of differential assignment due to unobserved confounders (84). The higher the gamma, the lower the sensitivity. Generally, if the gamma is greater than or equal to 2 and the *p*-value is significant, the results are insensitive to hidden bias (87).

As shown in Table 3, when gamma = 2, the treatment effects of regular physical exercise on physical health still is significant (*p* < 0.01). This finding indicated that this result was absolutely insensitive to hidden bias; when gamma = 1.8, the treatment effects of regular physical exercise on depression would no longer be significant (*p* = 0.08), which indicated that this result was possibly insensitive to hidden bias; and when gamma = 1.4, the treatment effects of regular physical exercise on cognitive health would no longer be significant (*p* = 0.97), which indicated that the result of the positive impact of regular physical exercise on cognitive health is not reliable.

**Table 3:** The results of the sensitivity analysis.

Gamma	Physical health		Emotional health		Cognitive health	
	Sig^+^	Sig^−^	Sig^+^	Sig^−^	Sig^+^	Sig^−^
1.0	0.000	0.000	0.000	0.000	0.000	0.000
1.1	0.000	0.000	0.000	0.000	0.000	0.000
1.2	0.000	0.000	0.000	0.000	0.000	0.000
1.3	0.000	0.000	0.000	0.000	0.000	0.017
1.4	0.000	0.000	0.000	0.000	0.000	0.965
1.5	0.000	0.000	0.000	0.000	0.000	10.000
1.6	0.000	0.000	0.000	0.003	0.000	10.000
1.7	0.000	0.000	0.000	0.019	0.000	10.000
1.8	0.000	0.000	0.000	0.077	0.000	10.000
1.9	0.000	0.000	0.000	0.211	0.000	10.000
2.0	0.000	0.000	0.000	0.833	0.000	10.000

Sig^+^ and Sig^−^ are the upper and lower limits of the significance level of a Wilcoxon signed-rank test, respectively.

## 4 Discussion

This longitudinal study is the first attempt to investigate the relationship between regular physical exercise among older adults and their emotional, physical, and cognitive health. The study applies the LPSM approach and utilizes data from two waves of the CFPS in 2010 and 2018. The present study aimed to address the significant public health concern of healthy aging, which has garnered increased attention globally.

Several specific findings emerged. The first major finding is that regular physical exercise among older adults was significantly associated with their physical health. Specifically, the scores of IADLs for older adults in the regular physical exercise group were 8.963% higher than those in the non-regular physical exercise group. This finding is in alignment with the findings from previous large-scale epidemiological studies, randomized controlled trials, and meta-analytic reviews (88–91), which demonstrated that regular physical exercise had a significant, beneficial association with the physical ability of older adults to carry out IADLs. Expanding and consolidating past research, this study was able to provide an accurate and compelling evidence for the positive role of regular physical exercise on physical function measures of IADLs by effectively eliminating sample-selection bias and mixed bias with the method of LPSM.

Second, in line with the previous studies (92–94), another key and consistent finding from this study was that, compared to older adults without physical exercise, older adults with regular physical exercise could be expected to have a lower level of depression. This finding is supported by the theory of social interaction (95). Based on this theory, social interactions that may facilitate relationships tends to bolster social relationships and thereby can be beneficial for reducing the level of depressive symptoms among older adults. As regular physical exercise in daily life could be a great relationship builder for older adults, it can also help promote their interpersonal communication skill and opportunity; consequently, these harmonious interpersonal relationships undoubtedly help old adults to relieve their depressive symptoms. On the other hand, long-term regular physical exercise may affect physiological health, which is conducive to improve depressive symptoms, such as an increasing level of monoamine transmitters, thereby restoring normal function of the hypothalamus–pituitary–adrenal axis (96, 97). Therefore, there is a need to encourage older adults with prior depressive illness, or at high risk of developing depressive illness, to participate in daily life physical exercise.

Third, although a statistically significant association was observed between regular physical exercise treatment and the cognitive health of older adults, the sensitivity analysis revealed that these results were sensitive to unobservable variables. This finding indicates that the potential positive association between regular physical exercise in later life and cognitive health may not be sufficient or valid. This result could explain the inconsistency across previous studies (98–100). For example, a review on physical exercise benefiting cognitive health during late periods of the lifespan found that 26 studies reported a positive correlation between physical exercise and maintenance or enhancement of cognitive health, five studies reported a dose–response relationship between physical activity and cognition, and one study showed a non-significant correlation (101). However, the majority of the evidence included in this review was of medium quality, and the overall risk of bias in the studies used in this review is moderately high (101). In future research, it is crucial to identify unobserved variables as potential confounding factors in order to improve the reliability of research conclusions.

Several limitations in this study need to be acknowledged in order to propose new directions for future investigations. First, the data used in this study are derived from the CFPS, which are second-hand survey data. These types of data have inherent limitations in terms of variable selection. Therefore, it is recommended that future research should develop specific research tools for assessing regular physical exercise, as well as physical, emotional, and cognitive health. Second, the covariates employed in this study may not encompass all the important potential confounding factors that could influence the relationship between regular physical exercise of older adults and their physical, emotional, and cognitive health. Factors such as drug use, life satisfaction, and intelligence level of older adults were not included in the analysis. Therefore, it is suggested that future research should investigate these factors as they could provide valuable insights. Third, although this study found a significant effect between regular physical exercise of older adults and their physical, emotional, and cognitive health, the sensitivity analysis revealed that the positive impact of regular physical exercise on cognitive health may not be entirely reliable. Consequently, future research should delve deeper into exploring the underlying mechanisms that link regular physical exercise with cognitive health. Fourth, it is important to note that regular physical exercise among older adults is a complex concept. This study only differentiated between older adults with regular physical exercise and sedentary older adults. Therefore, it did not investigate the specific types, frequencies, or intensities of regular physical exercise that may be most beneficial for self-care function, depression, and cognitive health of older adults. Future research should address this knowledge gap and provide more detailed insights into the optimal characteristics of regular physical exercise for older adults’ overall wellbeing.

The major empirical findings discussed above have important implications. First, policymakers should focus on creating effective and appropriate policies that promote enjoyable and lifelong physical exercise among older adults. These policies can provide formal and informal guidelines for communities to plan, implement, and evaluate physical exercise programs for older adults. Second, communities should offer a variety of age-appropriate sports and recreation programs that are appealing to older adults. It is also important to ensure that the spaces and facilities meet or exceed the recommended safety standards. Third, older adults need to recognize that they are more likely to engage in sedentary behavior, which can have negative health outcomes. Therefore, it is crucial for older adults to promote and participate in suitable physical activities to reduce sedentary time. Additionally, it is worth noting that physical activity levels tend to decline with age, and older adults often struggle to meet recommended guidelines. However, small interventions that encourage replacing sedentary activities with active ones can be effective. For example, incorporating more active travel, such as walking to nearby shops for small purchases, can be a feasible approach.

## 5 Conclusion

In conclusion, our study suggests that regular physical exercise may have a significant impact on reducing depressive symptoms and improving the physical function of older adults. However, we did not find sufficient evidence to suggest that it affects cognitive function, even when considering various confounding factors. These findings emphasize the importance of promoting regular physical exercise among older adults as part of active aging strategies, aiming to lessen the burden on public health.

## Data availability statement

The datasets presented in this study can be found in online repositories. The names of the repository/repositories and accession number(s) can be found below: The datasets generated and/or analyzed during the current study are available in the CFPS Public Data repository (www.isss.pku.edu.cn/cfps/en/) or from the corresponding author.

## Ethics statement

The studies involving humans were approved by the China Family Panel Studies (CFPS). CFPS was reviewed and approved by the Institute of Social Science Survey (ISSS) of Peking University (approval number: IRB00001052-14010). Respondents are given a statement explaining the purpose of the study, and all study participants had signed written informed consent prior to being investigated. The studies were conducted in accordance with the local legislation and institutional requirements. The participants provided their written informed consent to participate in this study.

## Author contributions

XX: Writing – original draft. YZ: Writing – original draft. JF: Writing – original draft. JH: Writing – original draft. XY: Writing – original draft. XZ: Writing – original draft. YL: Writing – review & editing. LC: Writing – review & editing. SW: Writing – review & editing.

## References

[1] Zhao Y Smith JP Strauss J. Can China age healthily? Lancet. (2014) 384:723–4. doi: 10.1016/S0140-6736(14)61292-7, PMID: 25176535 PMC4349321

[2] Beard JR Officer A de Carvalho IA Sadana R Pot AM Michel JP. The world report on ageing and health: a policy framework for healthy ageing. Lancet. (2016) 387:2145–54. doi: 10.1016/S0140-6736(15)00516-4, PMID: 26520231 PMC4848186

[3] Lopez-Otin C Blasco MA Partridge L Serrano M Kroemer G. The hallmarks of aging. Cell. (2013) 153:1194–217. doi: 10.1016/j.cell.2013.05.039, PMID: 23746838 PMC3836174

[4] Byrne C Faure C Keene DJ Lamb SE. Ageing, muscle power and physical function: a systematic review and implications for pragmatic training interventions. Sports Med. (2016) 46:1311–32. doi: 10.1007/s40279-016-0489-x, PMID: 26893098

[5] Goodpaster BH Park SW Harris TB Kritchevsky SB Nevitt M Schwartz AV. The loss of skeletal muscle strength, mass, and quality in older adults: the health, aging and body composition study. J Gerontol A Biol Sci Med Sci. (2006) 61:1059–64. doi: 10.1093/gerona/61.10.1059 17077199

[6] Donath L van Dieën J Faude O. Exercise-based fall prevention in the elderly: what about agility? Sports Med. (2016) 46:143–9. doi: 10.1007/s40279-015-0389-5, PMID: 26395115

[7] Morley JE Vellas B van Kan GA Anker SD Bauer JM Bernabei R. Frailty consensus: a call to action. J Am Med Dir Assoc. (2013) 14:392–7. doi: 10.1016/j.jamda.2013.03.022, PMID: 23764209 PMC4084863

[8] Ponti F Santoro A Mercatelli D Gasperini C Conte M Martucci M. Aging and imaging assessment of body composition: from fat to facts. Front Endocrinol. (2020) 10:861. doi: 10.3389/fendo.2019.00861, PMID: 31993018 PMC6970947

[9] Turner JE. Is immunosenescence influenced by our lifetime "dose" of exercise? Biogerontology. (2016) 17:581–602. doi: 10.1007/s10522-016-9642-z, PMID: 27023222 PMC4889625

[10] Turana Y Tengkawan J Chia YC Shin J Chen CH Park S. Mental health problems and hypertension in the elderly: review from the HOPE Asia network. J Clin Hypertens. (2021) 23:504–12. doi: 10.1111/jch.14121, PMID: 33283971 PMC8029564

[11] Alexopoulos GS. Depression in the elderly. Lancet. (2005) 365:1961–70. doi: 10.1016/S0140-6736(05)66665-2 15936426

[12] Tetsuka S. Depression and dementia in older adults: a neuropsychological review. Aging Dis. (2021) 12:1920–34. doi: 10.14336/AD.2021.0526, PMID: 34881077 PMC8612610

[13] Guhne U Stein J Riedel-Heller S. Depression in old age–challenge of an ageing society. Psychiatr Prax. (2016) 43:107–10. doi: 10.1055/s-0035-1552661 26158711

[14] Volkert J Schulz H Harter M Wlodarczyk O Andreas S. The prevalence of mental disorders in older people in Western countries - a meta-analysis. Ageing Res Rev. (2013) 12:339–53. doi: 10.1016/j.arr.2012.09.004, PMID: 23000171

[15] Evans DA. Estimated prevalence of Alzheimer's disease in the United States. Milbank Q. (1990) 68:267–89. doi: 10.2307/3350099 2233632

[16] Lu Y Liu C Yu D Fawkes S Ma J Zhang M. Prevalence of mild cognitive impairment in community-dwelling Chinese populations aged over 55 years: a meta-analysis and systematic review. BMC Geriatr. (2021) 21:10. doi: 10.1186/s12877-020-01948-3, PMID: 33407219 PMC7789349

[17] Dibben GO Dalal HM Taylor RS Doherty P Tang LH Hillsdon M. Cardiac rehabilitation and physical activity: systematic review and meta-analysis. Heart. (2018) 104:1394–402. doi: 10.1136/heartjnl-2017-312832, PMID: 29654095 PMC6109237

[18] Sun F Norman IJ While AE. Physical activity in older people: a systematic review. BMC Public Health. (2013) 13:449. doi: 10.1186/1471-2458-13-449, PMID: 23648225 PMC3651278

[19] Paluska SA Schwenk TL. Physical activity and mental health: current concepts. Sports Med. (2000) 29:167–80. doi: 10.2165/00007256-200029030-00003 10739267

[20] Mainguy V Provencher S Maltais F Malenfant S Saey D. Assessment of daily life physical activities in pulmonary arterial hypertension. PLoS One. (2011) 6:e27993. doi: 10.1371/journal.pone.0027993, PMID: 22110770 PMC3218075

[21] Gill JM Cooper AR. Physical activity and prevention of type 2 diabetes mellitus. Sports Med. (2008) 38:807–24. doi: 10.2165/00007256-200838100-00002 18803434

[22] LeBlanc AG Janssen I. Dose-response relationship between physical activity and dyslipidemia in youth. J Cardiol. (2010) 26:e201–5. doi: 10.1016/s0828-282x(10)70400-1, PMID: 20548982 PMC2903992

[23] Winzer EB Woitek F Linke A. Physical activity in the prevention and treatment of coronary artery disease. J Am Heart Assoc. (2018) 7:e007725. doi: 10.1161/JAHA.117.007725, PMID: 29437600 PMC5850195

[24] Gallanagh S Quinn TJ Alexander J Walters MR. Physical activity in the prevention and treatment of stroke. Int Schol. Res. Notices. (2011) 2011:2090–5513. doi: 10.5402/2011/953818, PMID: 22389836 PMC3263535

[25] McTiernan A Friedenreich CM Katzmarzyk PT Powell KE Macko R Buchner D. Physical activity in Cancer prevention and survival: a systematic review. Med Sci Sports Exerc. (2019) 51:1252–61. doi: 10.1249/MSS.0000000000001937, PMID: 31095082 PMC6527123

[26] Strohle A. Physical activity, exercise, depression and anxiety disorders. J Neural Transm (Vienna). (2009) 116:777–84. doi: 10.1007/s00702-008-0092-x 18726137

[27] de Oliveira L Souza EC Rodrigues RAS Fett CA Piva AB. The effects of physical activity on anxiety, depression, and quality of life in elderly people living in the community. Trends Psychiatry Psychother. (2019) 41:36–42. doi: 10.1590/2237-6089-2017-0129, PMID: 30994779

[28] Vancampfort D Stubbs B Veronese N Mugisha J Swinnen N Koyanagi A. Correlates of physical activity among depressed older people in six low-income and middle-income countries: a community-based cross-sectional study. Int J Geriatr Psychiatry. (2018) 33:e314–22. doi: 10.1002/gps.4796, PMID: 28994143

[29] Kim JH. Regular physical exercise and its association with depression: a population-based study short title: exercise and depression. Psychiatry Res. (2022) 309:114406. doi: 10.1016/j.psychres.2022.114406, PMID: 35074644

[30] Lampinen P Heikkinen R-L Ruoppila I. Changes in intensity of physical exercise as predictors of depressive symptoms among older adults: an eight-year follow-up. Prev Med. (2000) 30:371–80. doi: 10.1006/pmed.2000.0641 10845746

[31] Dziubek W Kowalska J Kusztal M Rogowski L Golebiowski T Nikifur M. The level of anxiety and depression in Dialysis patients undertaking regular physical exercise training--a preliminary study. Kidney Blood Press Res. (2016) 41:86–98. doi: 10.1159/000368548 26872253

[32] Portugal EMM Thais Cevada RSM-J Guimarães TT da Cruz E Rubini EL Charlene Blois ACD. Neuroscience of exercise: from neurobiology mechanisms to mental health. Neuropsychobiology. (2013) 68:1–14. doi: 10.1159/000350946, PMID: 23774826

[33] Liang J Wang H Zeng Y Qu Y Liu Q Zhao F. Physical exercise promotes brain remodeling by regulating epigenetics, neuroplasticity and neurotrophins. Rev Neurosci. (2021) 32:615–29. doi: 10.1515/revneuro-2020-0099, PMID: 33583156

[34] Paolucci EM Loukov D Bowdish DME Heisz JJ. Exercise reduces depression and inflammation but intensity matters. Biol Psychol. (2018) 133:79–84. doi: 10.1016/j.biopsycho.2018.01.015, PMID: 29408464

[35] Stathi A Fox KR McKenna J. Physical activity and dimensions of subjective well-being in older adults. J Aging Phys Activ. (2002) 10:76–92. doi: 10.1123/japa.10.1.76 18048943

[36] Cox EP O'Dwyer N Cook R Vetter M Cheng HL Rooney K. Relationship between physical activity and cognitive function in apparently healthy young to middle-aged adults: a systematic review. J Sci Med Sport. (2016) 19:616–28. doi: 10.1016/j.jsams.2015.09.003, PMID: 26552574

[37] Lü J Fu W Liu Y. Physical activity and cognitive function among older adults in China: a systematic review. J Sport Health Sci. (2016) 5:287–96. doi: 10.1016/j.jshs.2016.07.003, PMID: 30356530 PMC6188717

[38] de Souto BP Delrieu J Andrieu S Vellas B Rolland Y. Physical activity and cognitive function in middle-aged and older adults: an analysis of 104,909 people from 20 countries. Mayo Clin Proc. (2016) 91:1515–24. doi: 10.1016/j.mayocp.2016.06.032, PMID: 27720454

[39] Strhlein JK Bongard FVD Barthel T Reinsberger C. Dose-response-relationship between physical activity and cognition in elderly. Dtsch Z Sportmed. (2017) 2017:234–42. doi: 10.5960/dzsm.2017.300

[40] Loprinzi PD Edwards MK Crush E Ikuta T Del Arco A. Dose-response association between physical activity and cognitive function in a National Sample of older adults. Am J Health Promot. (2018) 32:554–60. doi: 10.1177/0890117116689732, PMID: 29214828

[41] Zheng P Pleuss JD Turner DS Ducharme SW Aguiar EJ. Dose-response association between physical activity (daily MIMS, peak 30-minute MIMS) and cognitive function among older adults: NHANES 2011-2014. J Gerontol A Biol Sci Med Sci. (2023) 78:286–91. doi: 10.1093/gerona/glac076, PMID: 35512348

[42] Miu DKY Szeto SL Mak YF. A randomised controlled trial on the effect of exercise on physical, cognitive and affective function in dementia subjects. Asian J Gerontol Geriatr. (2008) 3:8–16.

[43] Gates N Fiatarone Singh MA Sachdev PS Valenzuela M. The effect of exercise training on cognitive function in older adults with mild cognitive impairment: a meta-analysis of randomized controlled trials. Am J Geriatr Psychiatry. (2013) 21:1086–97. doi: 10.1016/j.jagp.2013.02.018 23831175

[44] Hernan MA Hernandez-Diaz S Robins JM. A structural approach to selection bias. Epidemiology. (2004) 15:615–25. doi: 10.1097/01.ede.0000135174.63482.43 15308962

[45] Djukanović I Sorjonen K Peterson U. Association between depressive symptoms and age, sex, loneliness and treatment among older people in Sweden. Aging Ment Health. (2015) 6:560–8. doi: 10.1080/13607863.2014.962001 25266255

[46] Luppa M Sikorski C Luck T Ehreke L Konnopka A Wiese B. Age- and gender-specific prevalence of depression in latest-life--systematic review and meta-analysis. J Affect Disord. (2012) 136:212–21. doi: 10.1016/j.jad.2010.11.033 21194754

[47] Moreno X Gajardo J Monsalves MJ. Gender differences in positive screen for depression and diagnosis among older adults in Chile. BMC Geriatr. (2022) 22:54. doi: 10.1186/s12877-022-02751-y, PMID: 35031004 PMC8760693

[48] Lillard LA Panis CW. Marital status and mortality: the role of health. Demography. (1996) 33:313–27. doi: 10.2307/2061764 8875065

[49] Zhang K Kan C Luo Y Song H Tian Z Ding W. The promotion of active aging through older adult education in the context of population aging. Front Public Health. (2022) 10:998710. doi: 10.3389/fpubh.2022.998710, PMID: 36299739 PMC9589353

[50] Zhu H Gu D. The protective effect of marriage on health and survival: does it persist at oldest-old ages? Journal of Population Ageing. (2010) 3:161–82. doi: 10.1007/s12062-011-9034-8

[51] Bulloch AGM Williams JVA Lavorato DH Patten SB. The depression and marital status relationship is modified by both age and gender. J Affect Disord. (2017) 223:65–8. doi: 10.1016/j.jad.2017.06.007, PMID: 28732242

[52] Rimashevskaia NM Kislitsyna OA. Income inequality and health. Sociol Methods Res. (2006) 45:43–62. doi: 10.1016/j.socscimed.2014.12.031

[53] Mishra S Carleton RN. Subjective relative deprivation is associated with poorer physical and mental health. Soc Sci Med. (2015) 147:144–9. doi: 10.1016/j.socscimed.2015.10.030, PMID: 26575605

[54] Reis Júnior WM Ferreira LN Molina-Bastos CG Bispo Júnior JP Reis HFT Goulart BNG. Prevalence of functional dependence and chronic diseases in the community-dwelling Brazilian older adults: an analysis by dependence severity and multimorbidity pattern. BMC Public Health. (2024) 24:140. doi: 10.1186/s12889-023-17564-w, PMID: 38200484 PMC10777626

[55] Antczak R Quashie NT Mair CA Arpino B. Less is (often) more: number of children and health among older adults in 24 countries. J Gerontol B Psychol Sci Soc Sci. (2023) 78:1892–902. doi: 10.1093/geronb/gbad123, PMID: 37622727 PMC10645313

[56] Xie Y Hu J. An introduction to the China family panel studies (CFPS). Chin Sociol Rev. (2014) 47:3–29. doi: 10.2753/CSA2162-0555470101.2014.11082908

[57] Xie Y Lu P. The sampling Design of the China Family Panel Studies (CFPS). Chin J Sociol. (2015) 1:471–84. doi: 10.1177/2057150X15614535, PMID: 29854418 PMC5973535

[58] Ke Y Jiang J Chen Y. Social capital and the health of left-behind older adults in rural China: a cross-sectional study. BMJ Open. (2019) 9:e030804. doi: 10.1136/bmjopen-2019-030804, PMID: 31772090 PMC6886947

[59] Su D Chen Z Chang J Gong G Guo D Tan M. Effect of social participation on the physical functioning and depression of empty-Nest elderly in China: evidence from the China health and retirement longitudinal survey (CHARLS). Int J Environ Res Public Health. (2020) 17:9438. doi: 10.3390/ijerph17249438, PMID: 33339258 PMC7766298

[60] Zhao Y Xu X Dupre ME Xie Q Qiu L Gu D. Individual-level factors attributable to urban-rural disparity in mortality among older adults in China. BMC Public Health. (2020) 20:1472. doi: 10.1186/s12889-020-09574-9, PMID: 32993592 PMC7526413

[61] Lawton MP Brody EM. Assessment of older people: self-maintaining and instrumental activities of daily living. The Gerontologist. (1969) 9:179–86. 5349366

[62] Tong A Man D. The validation of the Hong Kong Chinese version of the Lawton instrumental activities of daily living scale for institutionalized elderly persons. OTJR. (2002) 22:132–42. doi: 10.1177/153944920202200402

[63] Feng Z. Childlessness and vulnerability of older people in China. Age Ageing. (2018) 47:275–81. doi: 10.1093/ageing/afx137, PMID: 29096004 PMC6016684

[64] Lin S. Functional disability among middle-aged and older adults in China: the intersecting roles of ethnicity, social class, and urban/rural residency. Int J Aging Hum Dev. (2023) 96:350–75. doi: 10.1177/00914150221092129, PMID: 35422130 PMC9932620

[65] CFPS. China Family Panel Studies. (2010, 2018). Available online at: http://www.isss.pku.edu.cn/cfps/sjzx/gksj/index.htm (Accessed August 2, 2022).

[66] Huang G Xie Y Xu H. Cognitive ability: social correlates and consequences in contemporary China. Chin Sociol Rev. (2015) 47:287–313. doi: 10.1080/21620555.2015.1032161, PMID: 27570709 PMC4996474

[67] Jingwei H Yu X Chunni Z. The China family panel studies: design and practice. Society. (2014) 34:32.

[68] Qiong W Peihua L. Analysis of the reliability and validity of the mathematics test and vocabulary in the China family panel study. China Examin. (2016) 11:44–50.

[69] Qiong W Li P. Psychometric properties of the literacy test from China family panel studies. China Exam. (2016) 11:44–50. doi: 10.19360/j.cnki.11-3303/g4.2016.11.007

[70] Zhang X Chen X Zhang X. The impact of exposure to air pollution on cognitive performance. Proc Natl Acad Sci USA. (2018) 115:9193–7. doi: 10.1073/pnas.1809474115, PMID: 30150383 PMC6140474

[71] Li ZA-OX Qin WA-O Patel VA-O. Associations of parental depression during adolescence with cognitive development in later life in China: a population-based cohort study. PLoS Med. (2021) 18:e1003464. doi: 10.1371/journal.pmed.1003464, PMID: 33428637 PMC7799791

[72] Wang X Liu Y Zhao Z Liu W Chen Y Chen Y. Association of adolescent self-esteem in 2014 and cognitive performance in 2014, 2016, and 2018: a longitudinal study. Front Psychol. (2023) 14:14. doi: 10.3389/fpsyg.2023.1180397, PMID: 37205081 PMC10185744

[73] Radloff LS. The CES-D scale:a self-report depression scale for research in the general population. Appl Psychol Meas. (1977) 1:385–401. doi: 10.1177/014662167700100306

[74] Karim J Weisz R Bibi Z Rehman S. Validation of the eight-item center for epidemiologic studies depression scale (CES-D) among older adults. Curr Psychol. (2015) 34:681–92. doi: 10.1007/s12144-014-9281-y

[75] Bi K Chen P Chen S. Validating the 8-item Center for Epidemiological Studies Depression Scale-Chinese (CESD-Chinese). Data China Family Panel Stud. (2023). doi: 10.31219/osf.io/4brd7

[76] Liu JA-OX Qiang F Dang J Chen QA-O. Depressive symptoms as mediator on the link between physical activity and cognitive function: longitudinal evidence from older adults in China. Clin. Gerontologist. (2023) 46:808–18. doi: 10.1080/07317115.2022.2077158, PMID: 35603686

[77] Jie. Z, Zhen-Yun. W, Ge. F, Juan. L, Bu-Xin. H Zhi-Yan C. Development of the Chinese age norms of CES-D in urban area. Chin Ment Health J. (2010) 24:139–43.

[78] Dasso NA-O. How is exercise different from physical activity? A concept analysis. Nurs Forum. (2019) 54:45–52. doi: 10.1111/nuf.12296, PMID: 30332516

[79] van den Berg MH Schoones JW Vliet Vlieland TP. Internet-based physical activity interventions: a systematic review of the literature. J Med Internet Res. (2007) 9:e26. doi: 10.2196/jmir.9.3.e26, PMID: 17942388 PMC2047289

[80] Di Lorito C Long A Byrne A Harwood RH Gladman JRF Schneider S. Exercise interventions for older adults: a systematic review of meta-analyses. J Sport Health Sci. (2021) 10:29–47. doi: 10.1016/j.jshs.2020.06.003, PMID: 32525097 PMC7858023

[81] Pei Y Cong Z Wu B. Education, adult children's education, and depressive symptoms among older adults in rural China. Med J Soci Sci. (2020) 253:112966. doi: 10.1016/j.socscimed.2020.112966, PMID: 32247217

[82] Wang Y Zhang H Feng T Wang H. Does internet use affect levels of depression among older adults in China? A propensity score matching approach. BMC Public Health. (2019) 19:1474. doi: 10.1186/s12889-019-7832-8, PMID: 31699057 PMC6839058

[83] Silver IA Wooldredge J Sullivan CJ Nedelec JL. Longitudinal propensity score matching: a demonstration of counterfactual conditions adjusted for longitudinal clustering. J Quant Criminol. (2021) 37:267–301. doi: 10.1007/s10940-020-09455-9

[84] Rosenbaum PR Rubin DB. The central role of the propensity score in observational studies for causal effects. Biometrika. (1983) 70:41–55. doi: 10.1093/BIOMET/70.1.41

[85] Austin PC. An introduction to propensity score methods for reducing the effects of confounding in observational studies. Multivariate Behav Res. (2011) 46:399–424. doi: 10.1080/00273171.2011.568786, PMID: 21818162 PMC3144483

[86] Caliendo M Kopeinig S. Some practical guidance for the implementation of propensity score matching. J Econ Surv. (2008) 22:31–72. doi: 10.1111/j.1467-6419.2007.00527.x

[87] Rosenbaum PR. Sensitivity analysis for m-estimates, tests, and confidence intervals in matched observational studies. Biometrics. (2007) 63:456–64. doi: 10.1111/j.1541-0420.2006.00717.x, PMID: 17688498

[88] Roberts CE Phillips LH Cooper CL Gray S Allan JL. Effect of different types of physical activity on activities of daily living in older adults: systematic review and Meta-analysis. J Aging Phys Act. (2017) 25:653–70. doi: 10.1123/japa.2016-0201, PMID: 28181837

[89] Amaral Gomes ES Ramsey KA Rojer AGM Reijnierse EM Maier AB. The Association of Objectively Measured Physical Activity and Sedentary Behavior with (instrumental) activities of daily living in community-dwelling older adults: a systematic review. Clin Interv Aging. (2021) 16:1877–915. doi: 10.2147/CIA.S326686, PMID: 34737555 PMC8560073

[90] Zhou S Chen S Liu X Zhang Y Zhao M Li W. Physical activity improves cognition and activities of daily living in adults with Alzheimer's disease: a systematic review and Meta-analysis of randomized controlled trials. Int J Environ Res Public Health. (2022) 19:1216. doi: 10.3390/ijerph19031216, PMID: 35162238 PMC8834999

[91] Rydwik E Frandin K Akner G. Effects of a physical training and nutritional intervention program in frail elderly people regarding habitual physical activity level and activities of daily living--a randomized controlled pilot study. Arch Gerontol Geriatr. (2010) 51:283–9. doi: 10.1016/j.archger.2009.12.001 20044155

[92] Byeon H. Relationship between physical activity level and depression of elderly people living alone. Int J Environ Res Public Health. (2019) 16:4051. doi: 10.3390/ijerph16204051, PMID: 31652619 PMC6843978

[93] Awick EA Ehlers DK Aguiñaga S Daugherty AM Kramer AF McAuley E. Effects of a randomized exercise trial on physical activity, psychological distress and quality of life in older adults. Gen Hosp Psychiatry. (2017) 49:44–50. doi: 10.1016/j.genhosppsych.2017.06.005, PMID: 28662897 PMC5681423

[94] Hidalgo JL Sotos JR. Effectiveness of physical exercise in older adults with mild to moderate depression. Ann Fam Med. (2021) 19:302–9. doi: 10.1370/afm.2670, PMID: 34264835 PMC8282290

[95] Turner JH. A theory of social interaction. Stanford: Stanford University Press (1988).

[96] Kim YS O'Sullivan DM Shin SK. Can 24 weeks strength training reduce feelings of depression and increase neurotransmitter in elderly females? Exp Gerontol. (2019) 115:62–8. doi: 10.1016/j.exger.2018.11.009, PMID: 30453010

[97] Carneiro LS Mota MP Vieira-Coelho MA Alves RC Fonseca AM Vasconcelos-Raposo J. Monoamines and cortisol as potential mediators of the relationship between exercise and depressive symptoms. Eur Arch Psychiatry Clin Neurosci. (2017) 267:117–21. doi: 10.1007/s00406-016-0719-0, PMID: 27484978

[98] Jeong MK Park KW Ryu JK Kim GM Jung HH Park H. Multi-component intervention program on habitual physical activity parameters and cognitive function in patients with mild cognitive impairment: a randomized controlled trial. Int J Environ Res Public Health. (2021) 18:6240. doi: 10.3390/ijerph18126240, PMID: 34207701 PMC8296099

[99] Jia RX Liang JH Xu Y Wang YQ. Effects of physical activity and exercise on the cognitive function of patients with Alzheimer disease: a meta-analysis. BMC Geriatr. (2019) 19:181. doi: 10.1186/s12877-019-1175-2, PMID: 31266451 PMC6604129

[100] Ingold M Tulliani N Chan CCH Liu KPY. Cognitive function of older adults engaging in physical activity. BMC Geriatr. (2020) 20:229. doi: 10.1186/s12877-020-01620-w, PMID: 32616014 PMC7333382

[101] Carvalho A Rea IM Parimon T Cusack BJ. Physical activity and cognitive function in individuals over 60 years of age: a systematic review. Clin Interv Aging. (2014) 9:661–82. doi: 10.2147/CIA.S55520, PMID: 24748784 PMC3990369

