# Supplementary material for: Associations between regular physical exercise and physical, emotional, and cognitive health of older adults in China: an 8-year longitudinal study with propensity score matching
Source: Front Public Health. 2024 Apr 9;12:1301067. doi: 10.3389/fpubh.2024.1301067 (PMC11037100; doi:10.3389/fpubh.2024.1301067)
Supplement: Supplementary file 1 [file Table_1.docx]

**Supplementary Table 1**

*Correlation coefficients between covariates with independent variable and dependent variables*

| Covariates | Regular physical exercise | Physical  health | Emotional  health | Cognitive  health |
| --- | --- | --- | --- | --- |
| Gender | .070^**^ | .039 | -.162^**^ | .318^**^ |
| Age | .071^**^ | -.218^**^ | .035 | -.099^**^ |
| Education status | .374^**^ | .150^**^ | -.241^**^ | .750^**^ |
| Household registration | .501^**^ | .107^**^ | -.213^**^ | .459^**^ |
| Marital state | .033 | .063^**^ | -.175^**^ | .162^**^ |
| Number of children | -.084^**^ | -.055* | .004 | -.175^**^ |
| Income level | .100^**^ | .091^**^ | -.164^**^ | .019 |
| Chronic disease | .042 | -.112^**^ | .133^**^ | .065^**^ |
| Interpersonal relationships | .105^**^ | .104^**^ | -.160^**^ | .037 |
| Physical health^a^ | .096^**^ | .282^**^ | -.146^**^ | .122^**^ |
| Emotional health^a^ | -.251^**^ | -.163^**^ | .369^**^ | -.280^**^ |
| Cognitive health^a^ | .432^**^ | .185^**^ | -.253^**^ | .820^**^ |

Note: “a” represents variable’s data from 2010, the others from the data of 2018, ^*^*p*< .05, ^**^*p*< .01, ^***^ *p*< .001, the same below

**Supplementary Table 2**

*Descriptive characteristics of the total sample and differences between regular physical exercise group and non-physical exercise group before matching*

| Variables | Total sample  (N =1792) | | Regular physical exercise group  (N =761) | | Non-physical exercise group  (N =1031) | | Between regular physical exercise group and non-physical exercise group | | |
| --- | --- | --- | --- | --- | --- | --- | --- | --- | --- |
|  | Mean | SD | Mean | SD | Mean | SD | △Mean | *t* | *p* |
| Age | 73.660 | 4.876 | 74.060 | 5.039 | 73.365 | 4.732 | .696 | 2.993 | .003 |
| Education status | 1.959 | 1.999 | 2.829 | 2.035 | 1.316 | 1.709 | 1.513 | 17.073 | <.001 |
| Number of children | 2.628 | 1.393 | 2.491 | 1.292 | 2.728 | 1.455 | -.237 | -3.636 | <.001 |
| Income level | 2.989 | 1.205 | 3.129 | 1.088 | 2.886 | 1.275 | .243 | 4.346 | <.001 |
| Interpersonal relationships | 7.366 | 2.175 | 7.632 | 1.863 | 7.170 | 2.361 | .462 | 4.632 | <.001 |
| Physical health^a^ | 6.743 | .955 | 6.850 | .805 | 6.664 | 1.046 | .186 | 4.248 | <.001 |
| Emotional health^a^ | 1.508 | .685 | 1.308 | .502 | 1.658 | .760 | .347 | 11.632 | <.001 |
| Cognitive health^a^ | 19.550 | 16.110 | 27.649 | 15.206 | 13.571 | 14.015 | 14.078 | 20.270 | <.001 |
|  | **percent** |  | **percent** |  | **percent** |  | **△percent** | **χ^2^** | **p** |
|  | 0 | 1 | 0 | 1 | 0 | 1 | 1 |  |  |
| Gender | 46.931 | 53.069 | 42.838 | 57.162 | 49.952 | 50.048 | 7.114 | 8.895 | .003 |
| Household registration | 63.337 | 36.663 | 35.217 | 64.783 | 84.093 | 15.907 | 48.876 | 450.419 | <.001 |
| Marital status | 21.819 | 78.181 | 20.237 | 79.763 | 22.987 | 77.013 | 2.750 | 1.942 | .163 |
| Chronic disease | 68.136 | 31.864 | 65.834 | 34.166 | 69.835 | 30.165 | 4.001 | 3.228 | .041 |

Note: Gender: 0-female, 1-male; Marital status: 0-having no spouse, 1-having a spouse; Chronic disease: 0-having no chronic disease, 1-having at least one chronic disease.

**Supplementary Table 3**

*Differences between regular physical exercise group and non-physical exercise group after matching*

| Variables | regular physical exercise group  (N =428) | | Non-physical exercise group  (N =428) | | Between regular physical exercise group and Non-physical exercise group | | |
| --- | --- | --- | --- | --- | --- | --- | --- |
|  | Mean | SD | Mean | SD | △Mean | *t* | *p* |
| Age | 73.701 | 4.827 | 73.666 | 4.986 | .035 | .104 | .917 |
| Education status | 2.098 | 1.915 | 2.143 | 1.826 | -.044 | -.347 | .729 |
| Number of children | 2.631 | 1.363 | 2.626 | 1.444 | .005 | .049 | .961 |
| Income level | 3.028 | 1.181 | 3.012 | 1.182 | .016 | .202 | .840 |
| Interpersonal relationships | 7.521 | 1.963 | 7.528 | 2.119 | -.007 | -.050 | .960 |
| Physical health^a^ | 6.801 | .885 | 6.799 | .748 | .002 | .042 | .967 |
| Emotional health^a^ | 1.407 | .599 | 1.431 | .624 | -.024 | .568 | .570 |
| Cognitive health^a^ | 21.867 | 14.956 | 21.636 | 14.701 | .231 | .228 | .820 |
|  | percent |  | percent |  | △percent | χ2 | p |
|  | 0 | 1 | 0 | 1 | 1 |  |  |
| Gender | 51.527 | 48.473 | 47.945 | 52.055 | -3.582 | 1.075 | 0.333 |
| Household registration | 47.678 | 52.322 | 51.047 | 48.593 | 3.729 | 1.119 | 0.290 |
| Marital status | 49.698 | 50.302 | 51.031 | 48.969 | 1.333 | 0.107 | 0.744 |
| Chronic disease | 49.446 | 50.554 | 50.256 | 49.744 | .810 | 0.049 | 0.826 |
